# Supplementary material for: Some Aspects of Development and Histological Structure of the Visual System of Nothobranchius Guentheri
Source: Animals (Basel). 2021 Sep 21;11(9):2755. doi: 10.3390/ani11092755 (PMC8470241; doi:10.3390/ani11092755)
Supplement: Supplementary file 1 [file animals-11-02755-s001.zip › animals-1384846-supplementary.pdf]

**Table S1** – Primary data energy-dispersive X-ray spectroscopy (EDX) analysis lenses N. guentheri (Atomic Percentage).

| Sample name | C | O | Na | Mg | Al | Si | P | S | Cl | K | Ca | Fe | Ni | Cu | Zr | Summ. (at) |
|---|---|---|---|---|---|---|---|---|---|---|---|---|---|---|---|---|
| 2.1 | 73.57 | 25.25 | 0.11 | 0.16 | 0.40 | | 0.10 | 0.29 | 0.06 | | 0.04 | | 0.04 | | | 100.00 |
| 2.2 | 74.66 | 23.73 | 0.13 | 0.21 | 0.08 | 0.03 | 0.11 | 0.84 | 0.10 | | 0.07 | | | | 0.03 | 100.00 |
| 2.3 | 69.36 | 29.91 | 0.11 | 0.10 | 0.10 | 0.02 | 0.05 | 0.23 | 0.05 | | 0.03 | | 0.03 | | | 100.00 |
| 2.4 | 69.35 | 29.54 | 0.13 | 0.12 | 0.40 | 0.02 | 0.07 | 0.25 | 0.04 | | 0.04 | | 0.04 | | | 100.00 |
| 6.1 | 74.93 | 23.59 | 0.13 | 0.03 | 0.72 | | 0.04 | 0.43 | 0.03 | 0.02 | 0.05 | | 0.02 | 0.02 | | 100.00 |
| 6.2 | 72.65 | 26.65 | 0.09 | | 0.16 | 0.02 | 0.03 | 0.29 | 0.02 | | 0.03 | | 0.06 | | | 100.00 |
| 6.3 | 68.93 | 29.47 | 0.31 | 0.14 | 0.25 | | 0.06 | 0.52 | 0.02 | 0.02 | 0.19 | 0.01 | 0.07 | | | 100.00 |
| 6.4 | 68.44 | 29.69 | 0.18 | 0.20 | 0.88 | | 0.05 | 0.38 | 0.04 | | 0.08 | | 0.05 | | | 100.00 |

**Table S2** – Primary data energy-dispersive X-ray spectroscopy (EDX) analysis lenses N. guentheri (Weight Percent).

| Sample name | C | O | Na | Mg | Al | Si | P | S | Cl | K | Ca | Fe | Ni | Cu | Zr | Summ. (wt) |
|---|---|---|---|---|---|---|---|---|---|---|---|---|---|---|---|---|
| 2.1 | 66.80 | 30.54 | 0.18 | 0.29 | 0.81 | | 0.24 | 0.69 | 0.15 | | 0.12 | | 0.17 | | | 100.00 |
| 2.2 | 67.57 | 28.61 | 0.23 | 0.39 | 0.16 | 0.06 | 0.27 | 2.04 | 0.28 | | 0.22 | | | | 0.19 | 100.00 |
| 2.3 | 62.46 | 35.88 | 0.18 | 0.19 | 0.20 | 0.05 | 0.12 | 0.56 | 0.12 | | 0.09 | | 0.15 | | | 100.00 |
| 2.4 | 62.23 | 35.30 | 0.22 | 0.22 | 0.81 | 0.05 | 0.17 | 0.59 | 0.10 | | 0.11 | | 0.19 | | | 100.00 |
| 6.1 | 68.11 | 28.56 | 0.23 | 0.05 | 1.46 | | 0.09 | 1.05 | 0.08 | 0.05 | 0.14 | | 0.08 | 0.10 | | 100.00 |
| 6.2 | 66.03 | 32.27 | 0.15 | | 0.33 | 0.05 | 0.07 | 0.69 | 0.07 | | 0.09 | | 0.25 | | | 100.00 |
| 6.3 | 61.37 | 34.95 | 0.52 | 0.26 | 0.50 | | 0.13 | 1.23 | 0.06 | 0.06 | 0.58 | 0.05 | 0.29 | | | 100.00 |
| 6.4 | 60.84 | 35.16 | 0.31 | 0.37 | 1.75 | | 0.13 | 0.90 | 0.10 | | 0.23 | | 0.21 | | | 100.00 |

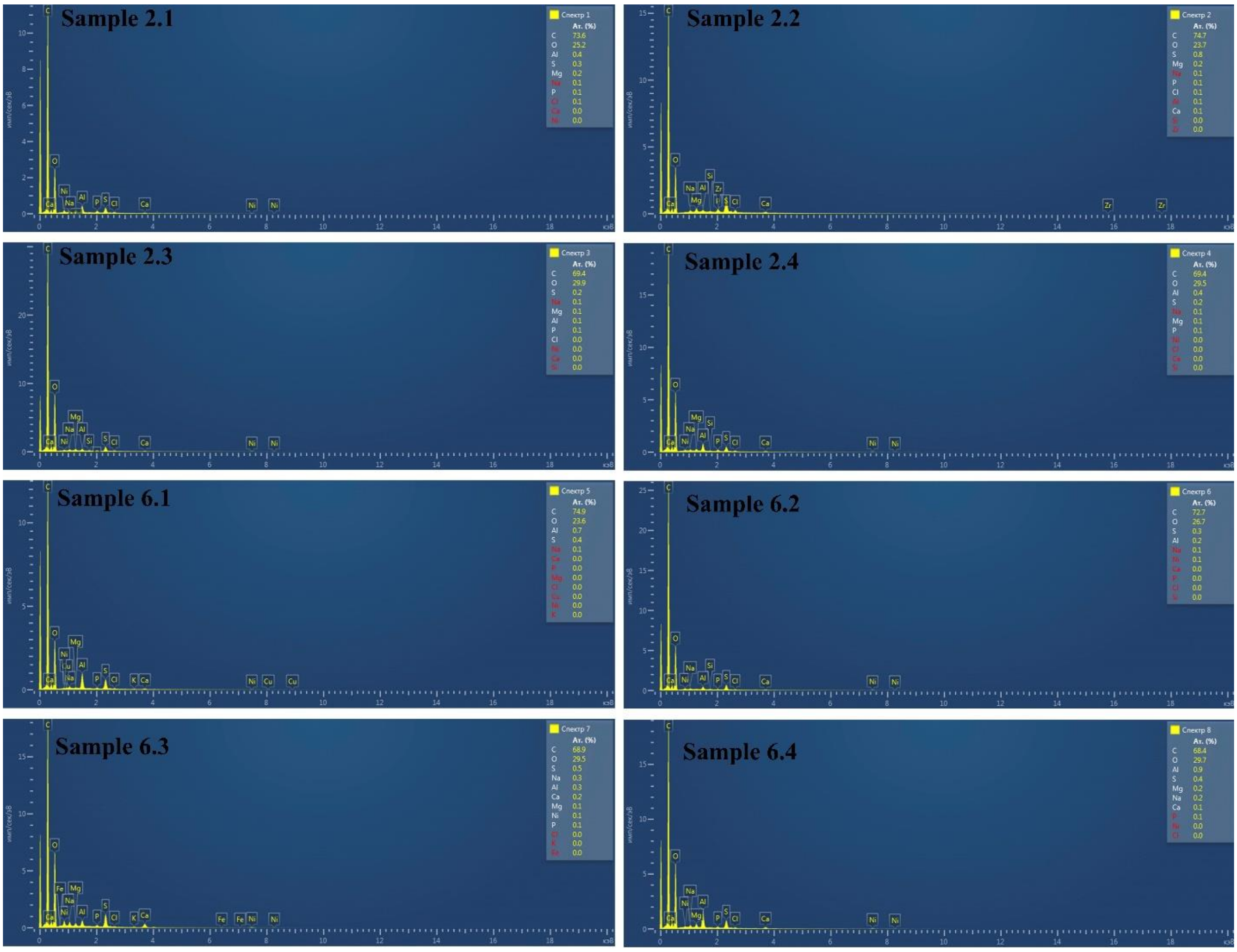


**Figure S1** - EDX-spectrum of lenses by group (mentioned in table S1, S2).

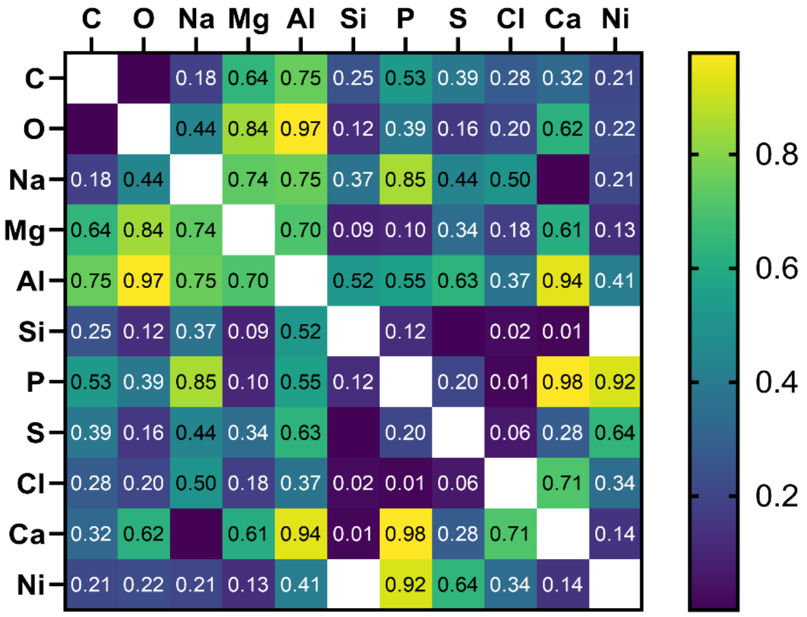


**Figure S2** - P values for Pearson Correlation as a correlation matrix between different elements in the lens.
